# Prevalence of disability in Manikganj district of Bangladesh: results from a large-scale cross-sectional survey

**DOI:** 10.1136/bmjopen-2015-010207

**Published:** 2016-07-18

**Authors:** Mohammad Moniruzzaman, M Mostafa Zaman, Saidur Rahman Mashreky, A K M Fazlur Rahman

**Affiliations:** 1 Noncommunicable Disease Unit, WHO Country Office for Bangladesh, Dhaka, Bangladesh; 2 Department of Public Health and Injury Prevention, Centre for Injury Prevention and Research Bangladesh, Dhaka, Bangladesh; 3 Department of Administration, Centre for Injury Prevention and Research Bangladesh, Dhaka, Bangladesh

**Keywords:** Disability, Prevalence, Bangladesh, Population

## Abstract

**Objective:**

To conduct a comprehensive survey on disability to determine the prevalence and distribution of cause-specific disability among residents of the Manikganj district in Bangladesh.

**Methods:**

The survey was conducted in Manikganj, a typical district in Bangladesh, in 2009. Data were collected from 37 030 individuals of all ages. Samples were drawn from 8905 households from urban and rural areas proportionate to population size. Three sets of interviewer-administered questionnaires were used separately for age groups 0–1 years, 2–10 years and 11 years and above to collect data. For the age groups 0–1 years and 2–10 years, the parents or the head of the household were interviewed to obtain the responses. Impairments, activity limitations and restriction of participation were considered in defining disability consistent with the International Classification of Functioning, Disability and Health framework.

**Results:**

Overall, age-standardised prevalence of disability per 1000 was 46.5 (95% CI 44.4 to 48.6). Prevalence was significantly higher among respondents living in rural areas (50.2; 95% CI 47.7 to 52.7) than in urban areas (31.0; 95% CI 27.0 to 35.0). Overall, female respondents had more disability (50.0; 95% CI 46.9 to 53.1) than male respondents (43.4; 95% CI 40.5 to 46.3). Educational deprivation was closely linked to higher prevalence of disability. Commonly reported prevalences (per 1000) for underlying causes of disability were 20.2 for illness, followed by 9.4 for congenital causes and 6.8 for injury, and these were consistent in males and females.

**Conclusions:**

Disability is a common problem in this typical district of Bangladesh, which is largely generalisable. Interventions at community level with special attention to the socioeconomically deprived are warranted.

**Strengths and limitations of this study**

- This study used a comprehensive definition of disability that is consistent with the International Classification of Functioning, Disability and Health framework for the first time in Bangladesh.
- The study was conducted in a typical district but selected purposively.
- Broad causes of disability (such as illness, congenital, environmental, injury) were identified, but further details could not be obtained except for injury.
- The representativeness of urban and rural population proportionate to size was considered.

## Introduction

Disability is universal and recognised as a global public health problem. People with disabilities face widespread barriers in accessing healthcare services, education, employment and social services, including housing and transport. They are also likely to experience social stigma, discrimination, inequality and disrespect, among many other hardships in their daily lives. These factors are the major reasons for their poorer health outcomes, lower educational achievement, lower economic participation, and higher rates of poverty than individuals without disabilities.^1^ ^2^ Globally, more than one billion people (or about 15% of the world's population) have been esti­mated to have some form of disability as reported in the first ever World Report on Disability,^2^ which clearly indicates that the prevalence of disability has risen since the 1970 estimate.^3^

Despite the high magnitude of disability cases, both awareness of and scientific information on disabilities are lacking in many countries such as Malawi, Mozambique, Zambia, Zimbabwe, India and Nepal.^2^ The lack of burden data may be due to the difficulty in obtaining this information given the complex nature and multidimensional characteristics. Because of this, many different approaches are used to collect the relevant data.^2^ Operational measures of disability vary widely across, and even within, countries. These variations occur because of the purpose and application of the data, the concept of disability, the aspects of disability examined (impairments, activity limitations, participation restrictions, related health conditions, environmental factors), the definitions, question design, reporting sources, data collection methods, and expectations of functioning.^2^

The magnitude of disability among Bangladeshi people is not precisely known. Data on disability burden are either not comprehensive^4^ and/or suffer from methodological limitations.^5^ ^6^ Recently, the Sample Vital Registration System (SVRS) and the Household Income and Expenditure Survey (HIES) have started reporting data on disabilities. The SVRS studies^7–9^ reported the prevalence to be ∼10 per 1000 of the Bangladeshi population. A non-governmental organisation reported a prevalence of five times that of the SVRS studies (56 per 1000).^10^ The HIES came up with a prevalence of 91 per 1000,^11^ which is nine times higher than the SVRS data. This study used the shorter version of the Washington Group Disability Questionnaire and did not cover children below 5 years of age. Presumably, the use of a comprehensive longer version of the Washington Group Questionnaire would yield a higher prevalence than that reported.

From the above picture, it is apparent that disability prevalence data for Bangladeshi people are extremely variable. The discrepancy of findings between studies needs to be resolved, and special focus is needed to define disability more comprehensively. Therefore we conducted this survey using a longer version of the Washington Group Questionnaire to describe the prevalence of disability in Bangladeshi people of all ages.

## Methods

A cross-sectional survey was conducted from July to December 2009 in the purposively selected Manikganj district of Bangladesh, which is located about 65 km north-west of the capital city, Dhaka. Manikganj is a typical district of Bangladesh with a central district town, seven moderately urbanised sub-districts with small townships (two of them municipalities), and a vast rural area including 1660 villages.^12^ It encompasses a population of 1.4 million with a population density of 1007/km^2^, a sex ratio (male/female) of 94.4, 20% of its inhabitants are living in urban areas, an average household (HH) size of 4.3 persons, and a literacy rate of 49.2% (for 7 years or older)—characteristics that closely resemble the demographic profile of the national population.^12^ ^13^

All non-institutional residents of all ages of the district were considered eligible for this survey. A total of 9450 HHs (2100 urban and 7350 rural proportionate to population size) were targeted to obtain a sample of 40 000 people. Using a prevalence of disability of 1% for Bangladesh (as estimated in the SVRS report) and 0.5% margin of error, we estimated that the minimum sample size required was 1521. This study aimed to estimate data in 10 groups according to gender, urban–rural area of residence and three age groups. Therefore, a minimum of 15 210 respondents were needed. To address the design effect (2.0) and potential response rate (76%), it was further inflated to a final sample size of 40 000.

In urban areas, 20 mahallas (the lowest urban geographic unit having identifiable boundaries) were selected randomly out of 64. From each mahalla, 105 HHs were selected to obtain our targeted number of HHs (2100). All members of the 105 selected HHs from each of the 20 mahallas constituted the urban samples (a total of 8800 individuals). Rural samples were also drawn from 10 randomly selected villages from each of the seven sub-districts (a total of 70 villages). From each village, 105 HHs were selected to obtain the targeted number of HHs (7350). All members of the selected HHs constituted the rural study sample (31 200 individuals). From each mahalla and village, 105 adjacent HHs starting from the randomly selected first HH in the middle of the selected cluster were visited to obtain the target number of HHs and individuals. This number (150 HHs) was calculated on the estimate of having an average HH size (4.2 persons) to obtain the targeted sample size in urban and rural areas. Ultimately, 37 030 (7293 urban and 29 737 rural) individuals from 8905 HHs were recruited. These constitute 94% and 93% response rates for HHs and individuals, respectively.

The field team underwent a 7-day training course before deployment. The team consisted of 20 experienced data collectors, five supervisors and one research manager. All had a masters degree in social sciences and prior experience in health-related research. The training was conducted by the Centre for Injury Prevention and Research, Bangladesh. Assistance from the local health authority was sought to ensure proper identification of sampling unit boundaries and cooperation of the local community. Data were collected by interviewer-administered questionnaires. We used three separate questionnaires for age groups 0–1 years, 2–10 years and 11 years or older. For the age groups 0–1 years and 2–10 years, the parents or the head of the HH were interviewed to obtain the responses. For the first two groups, we used disability questions from the Ten Question (TQ) Screen for Child Disability.^14^ ^15^ For the respondents aged 11 years or older, we took relevant questions from the first ‘Washington Group set of extended questions’ which was piloted under a project on Health and Disability Statistics by the United Nations Economic and Social Commission for Asia and the Pacific (UNESCAP) and WHO.^16^ We translated questions into Bangla as appropriate for the Bangladeshi context. Both forward and backward methodologies were applied during translation. In assigning disability, we applied two different approaches in the survey. Age-specific assessment of disability is discussed below.

- For the children aged 0–1 years and 2**–**10 years, the responses were ‘yes’ or ‘no’ for disability. In both cases, we used questions taken from the TQ Screen for Child Disability.^14^ ^15^ However, for children 0**–**1 years old, we dropped questions related to understanding and communication, as these questions were not appropriate for children less than 2 years old. For both age groups, the parents or the head of the HH were interviewed to obtain the responses. Variables researched for these age groups are given in boxes 1 and 2.
- For exploring disability among individuals aged 11 years or older, respondents were interviewed using 23 item questions under the six domains given in box 3.[Table BMJOPEN2015010207TB5]

Box 1 **Variables for 0–1 year age group (parents or household heads were interviewed)**

Do you think your child (mention the child's name) has the following health conditions/problems/difficulties compared with other children of the same age?

1. Delay in sitting, standing and walking.
2. Seeing difficulty either in the day time or at night.
3. Hearing difficulty (uses hearing aid, hears with difficulty, completely deaf).
4. Fits, becomes rigid, or loses consciousness (without fever).
5. Speaking difficulty (whether they can make themselves understood in words; can say any recognisable words).
6. Any abnormal behaviour (different from others/not generally acceptable).

Box 2 **Variables for 2–10 year age group (parents or household heads were interviewed)**

Do you think your child (mention the child's name) has the following health problems/difficulties compared with other children of the same age?

1. Delay in sitting, standing and walking.
2. Seeing difficulty either in the day time or at night.
3. Hearing difficulty (uses hearing aid, hears with difficulty, completely deaf).
4. Understanding when asked to do something.
5. Difficulty in walking or moving arms or weakness and/or stiffness in arms or legs.
6. Fits, becomes rigid, or loses consciousness (without fever).
7. Delay in learning something new (eg, poem, name of fruits/flowers/birds etc).
8. Speech is any way different (not clear enough to be understood by people other than the immediate family).
9. Appears in any way mentally backward, dull or slow.
10. Any abnormal behaviour (different from others/not generally acceptable).

Box 3 **Variables for respondents aged 11 years and older (respondents themselves were interviewed)**

**Table BMJOPEN2015010207TB5:** 

Domain 1: activity limitation due to health problems	Difficulties/clarifications/ examples
Visual problem	Seeing or recognising an object at arm's length or difficulty in seeing or recognising any known person across the road, even while wearing glasses
Hearing problem	Hearing what is said in conversation with one other person in a quiet room, even while wearing hearing aids
Problem with walking and climbing	Walking, climbing stairs or generally climbing up
Problem in memory/concentration	Remembering or concentrating on something
Problem in self-care	Self-care such as washing face and hands, bathing and other hygiene maintenance activities
Problem in performing activities of daily living	Completing necessary day-to-day activities such as: cleaning, cooking, home maintenance, shopping
Having any impairments	Existence of weakness of any body parts or loss of body parts or deformity or paralysis
Problem with communication	Communicating (eg, understanding or being understood by others)
Domain 2: body functions	
Having pain/discomfort	Discomfort, pains or aches in any part of the body
Hand function	Using hands and fingers—eg, to pick up objects, open containers
Domain 3: getting around	
Problem in standing	Standing for long periods such as 30 min or more
Sitting to standing	Changing position from sitting to standing
Moving inside home	Moving around and inside the home
Security outside home	Feeling insecure while outside of home
Walking long distances	Walking a long distance such as 1 km (or equivalent)
Domain 4: self-care	
Showering	Bathing or washing whole body
Dressing	Getting dressed
Eating	Eating or feeding oneself
Independent living	Living for a few days without another's assistance
Domain 5: understanding and communicating	
Concentration at work	Concentrating on doing something for 10 min or more
Memory loss	Forgetting to do important things
Domain 6: getting along with people	
Dealing with unknown person	Dealing with unknown people
Dealing with close person	Getting along with people who are close

### Disability measures

The prevalence of disability was calculated by taking the total number of participants who experience disability as the numerator and the total population screened for disability as the denominator. We used two separate approaches for defining disability. For children aged 0–1 and 2–10 years, the definition was decided on the basis of responses of parents or HH heads to the questions using the ‘yes’ or ‘no’ format (boxes 1 and 2). Any response in favour of physical or psychological limitation by the parent or HH head was identified as a case of disability.

For the respondents aged 11 years or older, we investigated everyone under the six domains with a total of 23 questions (box 3). Responses to these questions were categorised as follows: ‘not at all’ (recorded as 0); ‘just a little’ (recorded as 1); ‘moderate level’ (recorded as 2); ‘a lot of difficulty’ (recorded as 3); ‘unbearable’ (recorded as 4). We used flash cards to show the level of severity of the problem. Different colour flash cards were used for the different levels of severity. A case of disability was identified if all the responses in one domain received a score of ‘2’ or if any of the responses of any domain received a score of ‘3’ or ‘4’.

After identification of disability cases using the above ranking system and criteria, disability prevalence data in the three age groups, 0–14, 15–59 and 60 years or older, were presented. These age groups were selected for the purpose of international comparability, as they are used in the World Report on Disability 2011.^2^ We also calculated prevalence of the underlying causes of disability.

Prevalence was calculated per 1000 population with 95% CI. Categorical variables were presented as percentages. Data were analysed by SPSS software V.11.0. Prevalences were adjusted to the WHO world population.^17^

### Ethics considerations

Ethics approval was given by the Bangladesh Medical Research Council. We also obtained permission from the relevant administrative units of the surveyed district. This included the civil surgeon office, Upazila Health Complex and the local government office. Community leaders' (elected representatives of the local government offices such as municipality, upazila parishad and union council) orientations were done before starting the survey for their participation in its implementation process. Finally, verbal consent from individual respondents was obtained. Assent in the case of children aged 0–10 years was obtained from their parents.

## Results

Almost half (49.9%) of the respondents were male, and one-fifth (19.7%) were from urban areas. Overall, nearly two-thirds (62.6%) of the respondents were of working age (15–59 years), and less than one-third (29.8%) were from the youngest age group (0–14 years). Only 7.6% were from the oldest age group (60 years or older). The median age of the respondents was 26 years in both urban and rural areas. About a quarter of the respondents (22.5%) had received no formal education, nearly one-third (31.2%) had received some primary education, and just over a quarter (26.7%) had received some secondary education. Only 7.9% had received education at the higher secondary and above level. Respondents aged 6 and below constituted 11.7% of the sample and were not relevant in this section. The most commonly reported occupations were housewife (33.3%), student (26.0%), agriculture (10.2%), business (8.0%) and employed (7.8%) as shown in table 1.

**Table 1:** Sociodemographic characteristics of the survey population

Variable	Urban (n=7293)	Rural (n=29 737)	Total (n=37 030)
Age group (years), %			
0–14	29.5	29.8	29.8
15–59	64.1	62.2	62.6
60 and above	6.4	8.0	7.6
Age (years), median (IQR)	26.9 (12.7–40.8)	26.0 (12.3–42.0)	26.1 (12.4–41.8)
Sex, %			
Male	50.3	49.9	49.9
Female	49.7	50.1	50.1
Educational level, %			
No formal education	18.2	23.6	22.5
Any primary (1–5 years)	28.8	31.7	31.2
Any secondary (6–10 years)	28.5	26.2	26.7
Higher secondary and above (11 years and above)	13.3	6.6	7.9
Not applicable (below 6 years)	11.2	11.9	11.7
Occupation*, %			
Agriculture	4.8	11.5	10.2
Business	10.5	7.4	8.0
Student	26.8	25.8	26.0
Housewife	32.3	33.1	33.0
Employed	9.9	7.3	7.8
Unemployed	3.1	4.1	3.9
Labourer	6.5	6.1	6.2
Retired	2.4	2.3	2.3
Other	3.7	2.4	2.6

*Excluding 4339 aged below 6 years.

Our sex-specific analysis showed that, among the female respondents, the commonly reported occupations were housewife (65.1%), student (25.5%) and employed (2.1%). For the male respondents, the commonly reported occupations were student (26.5%), agriculture (20.0%), business (15.7%), employed (13.5%) and labour (11.2%).

For measuring disability among respondents aged 11 years or older, we used a comprehensive questionnaire consisting of 23 questions grouped under six domains. Responses recorded as ‘just a little’ problem (per 1000 population) ranged from 3.4 to 215.8, ‘moderate level’ from 1.8 to 13.2, ‘a lot of difficulty’ from 0.8 to 3.6, and ‘unbearable’ from 0.3 to 3.5. Commonly reported difficulties were related to body pain/discomfort, sitting to standing, seeing, walking/climbing, standing for at least 30 min, weak/paralysis, or loss of body part (table 2).

**Table 2:** Distribution of domain-specific problems (per 1000 population) among respondents aged ≥11 years (n=28 818)

	Response				
Domain	Not at all (score=0)	Just a little (score=1)	Moderate level (score=2)	Lot of difficulty (score=3)	Unbearable (score=4)
1. Activity limitation due to health problems					
Seeing	874.8	110.2	12.1	2.2	0.7
Hearing	965.1	28.9	4.5	1.0	0.4
Walking/climbing	880.5	104.0	11.7	2.2	1.6
Memory/concentration	978.5	17.0	3.4	0.8	0.3
Self-care	983.6	8.2	5.3	1.4	1.5
Daily activity	922.0	62.9	11.0	2.2	2.0
Weak or loss of body part	912.8	69.4	13.2	3.6	1.1
Communication	988.4	6.3	3.5	1.2	0.6
2. Body function (last 30 days)					
Body pain/discomfort	772.4	215.8	10.1	1.4	0.3
Hand function	990.8	4.3	3.0	0.9	1.0
3. Getting around (last 30 days)					
Standing (at least 30 min)	908.6	76.2	10.8	2.2	2.2
Sitting to standing	859.6	128.9	8.2	1.5	1.9
Moving inside home	983.4	9.6	4.2	1.2	1.6
Security outside home	981.7	8.3	6.1	2.1	1.7
Walking equivalent to 1 km	900.3	83.3	9.8	3.1	3.5
4. Self-care (last 30 days)					
Showering	984.8	7.1	5.0	1.0	2.2
Dressing	989.7	4.3	3.5	1.0	1.6
Eating	992.8	3.4	1.8	0.8	1.2
Independent living	979.7	11.6	4.5	1.6	2.6
5. Understanding and communicating (last 30 days)					
Concentration at work for 10 min	989.5	6.5	2.7	0.9	0.4
Memory loss for important things	969.7	25.1	3.6	0.9	0.6
6. Getting along with people (last 30 days)					
Dealing with unknown person	986.0	8.7	3.3	1.4	0.6
Dealing with close person	992.4	4.4	1.9	0.9	0.3

After ascertainment of all disability cases from all respondents, we calculated the crude prevalence of disability and then adjusted it to the WHO standard population. The overall age-standardised prevalence of disability among the respondents was 46.5 per 1000 population. In rural areas, it was significantly higher (50.2) than in urban areas (31.0). Overall, the prevalence was higher among female (50.0) than male (43.4) respondents. The oldest age group (≥60 years) had the highest prevalence (164.3) overall, as shown in table 3.

**Table 3:** Prevalence of disability per 1000 population (95% CI) (n=37 030)

Sex	Age* (years)	Urban	Rural	Overall
Male				
	0–14	17.9 (10.1 to 25.7)	39.9 (34.1 to 45.7)	35.5 (30.6 to 40.4)
	15–59	17.8 (12.4 to 23.2)	31.8 (28.2 to 35.4)	29.0 (25.9 to 32.1)
	≥60	102.5 (64.4 to 140.6)	142.1 (122.4 to 161.8)	135.5 (117.9 to 153.1)
	All ages (crude)	23.5 (18.6 to 28.4)	43.2 (39.9 to 46.5)	39.3 (36.5 to 42.1)
	All ages (standardised)†	27.9 (22.6 to 33.2)	47.1 (43.7 to 50.5)	43.4 (40.5 to 46.3)
Female				
	0–14	18.4 (10.2 to 26.6)	34.0 (28.7 to 39.3)	31.1 (26.5 to 35.7)
	15–59	15.2 (10.3 to 20.1)	33.5 (29.8 to 37.2)	29.8 (26.7 to 32.9)
	≥60	166.9 (118.0 to 215.8)	200.2 (177.1 to 223.2)	195.6 (174.6 to 216.6)
	All ages (crude)	25.4 (20.3 to 30.5)	46.6 (43.2 to 50.0)	42.4 (39.5 to 45.3)
	All ages (standardised)†	34.2 (28.3 to 40.1)	53.6 (50.0 to 57.2)	50.0 (46.9 to 53.1)
Both				
	0–14	18.2 (12.5 to 23.9)	37.0 (33.1 to 40.9)	33.3 (29.9 to 36.7)
	15–59	16.5 (12.8 to 20.2)	32.6 (30.0 to 35.2)	29.4 (27.2 to 31.6)
	≥60	133.8 (102.9 to 164.7)	170.5 (155.3 to 185.7)	164.3 (150.7 to 177.9)
	All ages (crude)	24.4 (20.9 to 27.9)	44.9 (42.5 to 47.3)	40.9 (38.9 to 42.9)
	All ages (standardised)†	31.0 (27.0 to 35.0)	50.2 (47.7 to 52.7)	46.5 (44.4 to 48.6)

*Age grouping according to the World Disability Report 2011.

**†**Standardised to the age distribution of the new WHO standard population (2000–2025).

We also calculated the prevalence of the underlying causes of disability. The top underlying cause of disability per 1000 population was illness (20.2) followed by congenital causes (9.4) and injury (6.8). These were more or less consistent across gender in general (table 4).

**Table 4:** Prevalence of underlying causes of disability per 1000 population

Sex	Urban	Rural	Overall
Male	(n=3667)	(n=14 839)	(n=18 506)
Injury*	6.5	9.0	8.5
Congenital	4.6	10.7	9.5
Illness†	10.9	20.1	18.3
Other causes	1.4	3.4	3.0
Total	23.4	43.2	39.3
Female	(n=3626)	(n=14 898)	(n=18 524)
Injury	2.5	5.9	5.1
Congenital	5.8	10.5	9.3
Illness	13.8	25.1	22.2
Other causes	3.3	6.7	5.8
Total	25.4	48.2	42.4
Both	(n=7293)	(n=29 737)	(n=37 030)
Injury	4.5	7.4	6.8
Congenital	5.2	10.4	9.4
Illness	12.3	22.2	20.2
Other causes	2.3	4.9	4.5
Total	2.3	4.9	4.5

*Physical harm or damage to body resulting from external mechanical force, drowning, poisoning, fall or burns, violence from assault, and self-inflicted violence.

†A condition of being unhealthy in body or mind due to sickness or disease.

**Figure 1:**
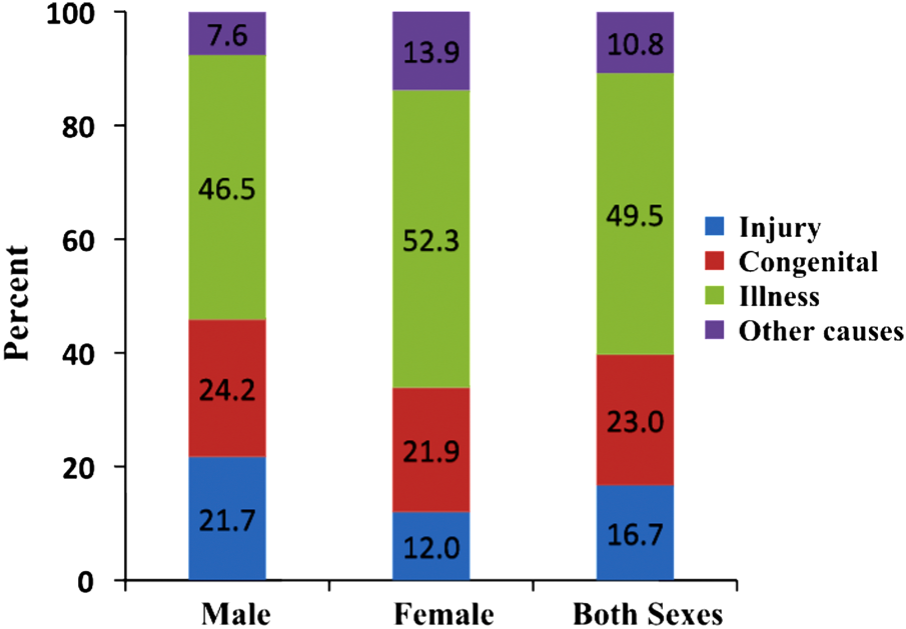
Proportion of causes of disability (n=1513).

The proportion of underlying causes of disability was also looked at. The most common cause was illness, which was reported by half of the respondents (49.5%), and was found more commonly in female (52.3%) than male (46.5%) respondents. The second most common cause was congenital, reported by about a quarter (23.0%) of the respondents and evenly distributed among male and female respondents. The third most common cause was injury, reported by about one-sixth (16.7%) of the respondents and almost twice as common in male (21.7%) as female (12.0%) respondents (figure 1).

**Figure 2:**
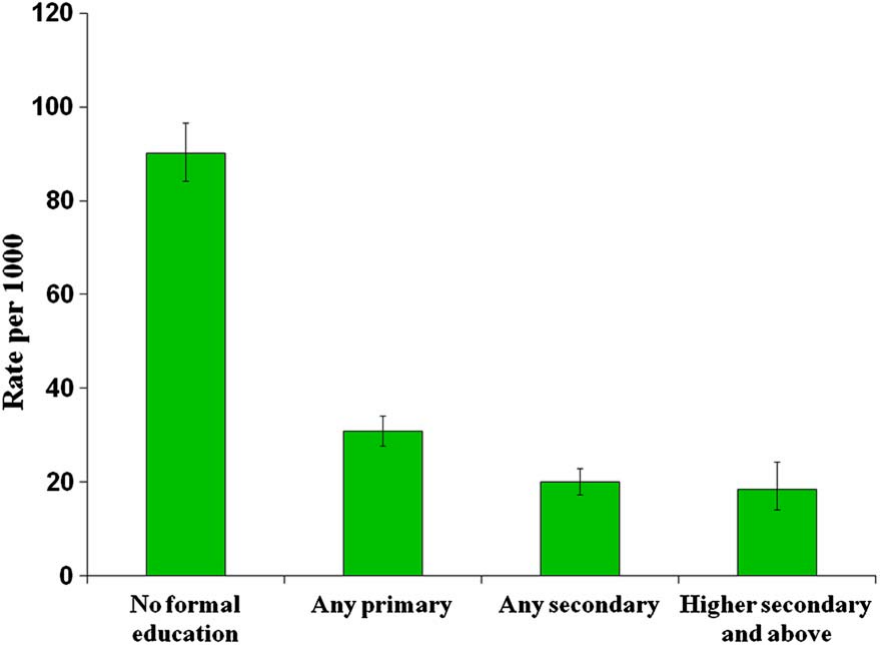
Prevalence of disability by education level (rate per 1000). Error bars indicate CIs.

We also investigated the prevalence of disability according to the respondents' level of education. As shown in figure 2, we found that people with disability had significantly less formal education than the general population. Rates (per 1000 population) were: no formal education, 90.1 (95% CI 84.1 to 96.5); any primary education, 30.8 (27.8 to 34.2); any secondary education 20 (17.4 to 23); higher secondary education and above, 18.5 (14.1 to 24.3).

## Discussion

This survey focused on the prevalence of disability. We conducted a dedicated survey using more in-depth questions to generate more comprehensive information on disability. The survey was conducted in a particular district, Manikganj, to provide accurate and disaggregated data on prevalence of disability and the distribution of causes that resulted in permanent disability.

Our prevalence estimate of disability is 46.5 per 1000 population or 4.7%, which is comparable with many countries in the Asia-Pacific Region where disability prevalence ranges from 1.0% to 18.6% with a mean prevalence of 4.6% estimated by UNESCAP in 2012.^18^ However, it is far below the global prevalence of disability (15.0%) estimated jointly by the WHO and the World Bank.^2^ We also observed, from the UNESCAP report,^18^ that sub-regional disability prevalences vary widely. The average disability prevalence ranges from 2.6% in South-East Asia to 17.0% in the Pacific. In the same report, the disability prevalence in Bangladesh is reported to be 0.9%, referring to the SVRS report (9 per 1000 population).^7^

The disability prevalence determined in the present study is similar to that in our neighbouring country, India (6.3%), which was estimated in a community-based study conducted in a rural area.^19^ In contrast, it was reported to be as low as 2.0% in India by the National Sample Survey Organization^20^ and Census data.^21^ A review article by Kumar *et al*^22^ stated that disability prevalences vary in India.

The variation in disability prevalences at regional level, among countries, and within a country is also reflected in the World Report on Disability 2011.^2^ We observed this variation in Bangladesh also. The prevalence we found is lower by half than that (9.1%) reported in HIES 2010 for Bangladesh^11^ and that (10.5%) reported in another study carried out to field test the rapid assessment of a disability questionnaire among adults aged ≥18 years old in Bangladesh.^23^ It also contrasts with the prevalence of 9 per 1000 population or 0.9% reported in the SVRS Survey 2013.^9^ These variations might be explained by differences in operational measures of disability, designing question, reporting sources, data collection methods, and the purpose and application of the data. These factors are common in both developed and developing countries.^2^ ^18^ ^22^ In our study, respondents living in a rural area, women, older people, and people with lower educational achievement are more likely to report having disabilities. These findings are similar to other available data in Bangladesh^11^ ^24^ and in common with those obtained in many other countries.^2^

This study has some limitations. We measured disability on the basis of subjective responses given by either the parents or HH heads for children aged 0–10 years. For those aged 11 years and above, the measurement was made according to the respondents' own answers. This method of data collection might influence the final estimate because of the possibility of under- or over-estimating. Physical or medical examinations in certain contexts could serve as a more reliable means of verification of certain health-related conditions, hearing and visual problems, presence of any impairment that may lead to disability. We used a visual analogue scale to measure the severity of problems for all respondents aged 11 years or older, which might have led to over- or under-estimation of the severity score. This can be explained by variability in individual understanding, level of education and gender. We collected information on causes of disability under broad headings of illness, congenital and environmental, etc. It would have been better to have detailed information on causes of disability for the purpose of tailored intervention. We had detailed information only for injury.

Given that disability is a complex multidimensional condition, acquiring burden data presents numerous challenges.^2^ Therefore, we used context-, age- and domain-specific questionnaires to measure disability. We included people of all ages and used three separate questionnaires that were adapted from widely used valid tools for three selected age groups in order to capture accurate disability measures. We ensured representative sampling from urban and rural settings. The demographic profile of our surveyed district is also highly consistent with the national population. Therefore, it can be claimed that the prevalence estimate from this study represents the national level.

## Conclusion

Disability is a common problem in this typical district of Bangladesh, which is largely generalisable. Interventions at community level with special attention to the socioeconomically deprived are warranted. Interventions targeting commonly reported causes (illness, congenital and injury) will be instrumental in reducing the number of victims and consequences of avoidable disability. However, further study is required to detail the causes of disability.
